# Two Rare Cases of Hepatocellular Carcinoma after Kasai Procedure for Biliary Atresia: A Recommendation for Close Follow-Up

**DOI:** 10.1155/2015/982679

**Published:** 2015-08-03

**Authors:** Alicia C. Hirzel, Beatrice Madrazo, Claudia P. Rojas

**Affiliations:** Department of Pathology, Jackson Memorial Hospital, University of Miami Miller School of Medicine, 1611 NW 12th Avenue, Holtz Building, Room 2042, Miami, FL 33136, USA

## Abstract

The instigation of the Kasai procedure in infants who are born with biliary atresia has led to increased survival in this population for over half a century. The many complications that arise as a result of biliary atresia led to an early death for most patients. However, the Kasai procedure is not without its own impediments. Among them is the development of hepatocellular carcinoma. We present two cases of hepatocellular carcinoma, after Kasai procedure, from two different age groups, as a recommendation that these patients should be even more closely monitored. Furthermore, if they are in need of transplant, we recommend that the explanted livers be carefully examined, as the tumor may not have been diagnosed preoperatively.

## 1. Introduction

Biliary atresia is an idiopathic obliteration of the extra- and intrahepatic biliary system leading to the obstruction of bile flow. The etiology of biliary atresia is unknown and its pathogenesis is not clearly understood. However, if left untreated, the cholestasis results in cirrhosis and liver failure. It is one of the many causes of neonatal cholestasis. The incidence varies from region to region with the highest occurring in the Pacific at 1 in 2,400 live births [1].

Biliary atresia may be syndromic or nonsyndromic [2]. Ultrasound may be performed and shows a shrunken gallbladder despite a 12-hour fast. Cholangiography assesses the morphology and patency of the gallbladder and ducts. The histologic findings in each type are similar to liver biopsy generally showing bile plugs, ductular proliferation, and fibrosis with obliteration of the ducts. These along with liver function tests help to confirm the diagnosis.

Currently, patients are managed surgically with the Kasai operation early in life. The goal of the procedure is to restore bile flow. In 1951, Dr. Morio Kasai developed the “Kasai” procedure or hepatoportojejunostomy or “Roux-en-Y.” This procedure bypasses the bile ducts and allows the bile to drain into the intestines, thus slowing the liver damage. The complications of biliary atresia stem from the development of progressive cirrhosis. The complications of the Kasai surgery include cholangitis, portal hypertension, hepatopulmonary syndrome, pulmonary hypertension, biliary cysts, and malignancies such as hepatoblastomas, hepatocellular carcinomas, and cholangiocarcinomas. While they do occur, hepatocellular carcinomas (HCCs) are rare entities after Kasai procedure and only few cases are reported.

Here we present two cases of hepatocellular carcinoma after Kasai procedure for biliary atresia.

## 2. Case 1

**Figure 1:**
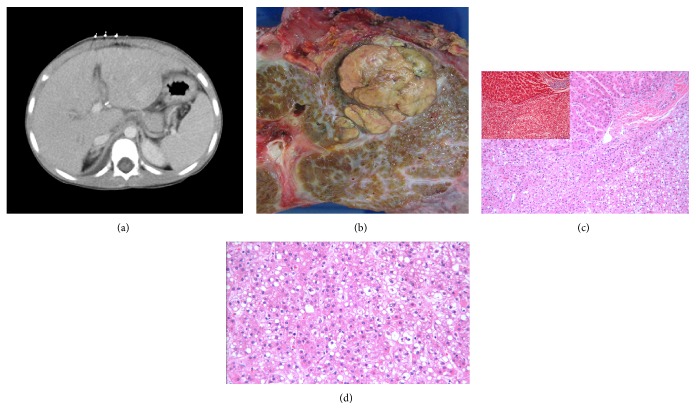
(a) CT scan of liver. (b) Gross picture of liver mass. (c) Microscopic picture of liver mass, H&E, 10x, with trichrome inset showing mass in lower half. (d) High magnification of mass, H&E, 20x.

A 4-year-old boy, delivered via C-section, presented with obstructive jaundice, dark urine, and clay-colored stool soon after birth (after an uneventful gestational period). The liver biopsy was interpreted as cirrhotic liver with cholestasis, bile ductular proliferation, and bile lakes, consistent with extrahepatic biliary atresia. He suffered an episode of cholangitis prior to his Kasai procedure, which was then postponed until after treatment. He was then lost to follow-up. At 4 years of age he was taken to the hospital due to an enlarging abdominal mass. Ultrasound and magnetic resonance imaging (MRI) detected two lesions, one in segment II of the liver with a second smaller mass in segment III (Figure 1(a)). Biopsies proved the masses to be hepatocellular carcinoma. Liver transplant was scheduled shortly thereafter. The explanted liver had two nodules in the left lobe, 3.8 cm and 2 cm, seen grossly, which were diagnosed as well-differentiated hepatocellular carcinoma and staged as pT2pNx (Figure 1(b)). Reticulin stain disclosed disruption of the lining around the hepatocytes in the hepatocellular carcinoma. Trichrome also highlighted areas of neoplastic tissue (Figures 1(c) and 1(d)). The patient presented with acute rejection 5 months after transplant and was treated. At 8 months after transplant, a liver biopsy was performed and chronic rejection with distortion and paucity of bile ducts was diagnosed. One year after transplant another biopsy was performed which showed bile duct proliferation with cholestasis, parenchymal inflammation, and portal fibrosis. Immunostain for SV40 was negative as was polymerase chain reaction (PCR) for BK virus. After this last biopsy the patient was again lost to follow-up and has not returned to our institution.

## 3. Case 2

**Figure 2:**
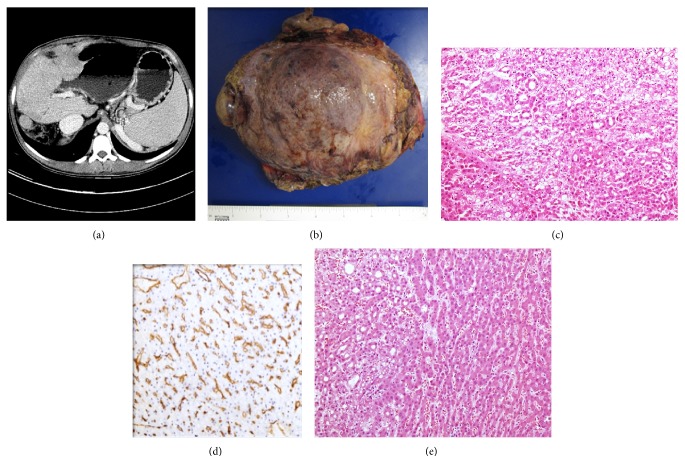
(a) CT scan of liver. (b) Gross picture showing nodular surface with somewhat exophytic mass on right. (c) Microscopic picture of mass, H&E, 20x. (d) Main mass with immunohistochemical stain for CD34 outlining neovascularization. (e) H&E of second “subnodule,” 10x.

A 25-year-old physician underwent a two-step Kasai procedure as an infant, first at 40 days then at 2 years of age. Surgeries were performed in another country. He had not received any surveillance endoscopies for varices since he was about 5 years of age. His clinical course was complicated by splenomegaly, thrombocytopenia, nonbleeding esophageal varices, and hepatic encephalopathy mostly due to the progression to cirrhosis. The varices regressed and his treating physician in his native country believed that he developed shunts that contributed to development of his encephalopathy. A CT liver triple phase with contrast showed a cirrhotic liver with small hypodensity and portal venous phase that persisted on delayed images in the right inferior lobe (Figure 2(a)). The patient underwent liver transplant. Two nodules were seen in the explanted liver, both in segment 7, measuring 2.5 and 1.2 cm in greatest dimension. The nodules were positive for polyclonal CEA, CD10, arginase-1, glypican 3 (focal and granular), CD34 (Figure 2(d)), and beta-catenin (membranous). They were negative for alpha-fetoprotein immunostain. The larger nodule was encapsulated and exophytic in nature (Figure 2(b)). The smaller nodule was a “subnodule” growing within a high-grade dysplastic nodule (Figure 2(e)). They were both diagnosed as well-differentiated hepatocellular carcinoma and staged as pT2pNX. Approximately 2 months after transplant, a liver biopsy was done due to persistently elevated transaminases. However, no acute rejection was seen. The patient returned to his native country shortly thereafter.

## 4. Discussion

Hepatocellular carcinoma (HCC), while not an unknown entity, is a rare occurrence after Kasai procedure, especially in the adult and neonatal population. Hol et al. reported a case in a 19-year-old individual while Aggarwal et al. reported another case in an older individual [3, 4]. It is also known to be very rare in infants under the age of 1 [5, 6]. HCC is more prone to develop in cirrhotic livers and in patients with biliary atresia. In addition, dysplastic nodules have been described in cirrhotic explanted livers [7, 8]. Our second patient had hepatocellular carcinoma developing in a dysplastic nodule. Here we describe two cases of well-differentiated hepatocellular carcinoma in patients who underwent Kasai procedure due to biliary atresia. The timing of the development of the malignancies was different in each patient with the first showing symptoms at 4 years of age while the second was diagnosed at 24 years of age, after examination of his explanted liver. The nodules in this second case were not seen in imaging prior to transplant and, thus, were an unexpected finding.

There are three types of biliary atresia depending on anatomic findings. Type I involves only the common bile duct (CBD) while the gallbladder and the hepatic ducts are patent. Type II involves the hepatic duct only with patent proximal intrahepatic ducts and is further classified into IIa (patent gallbladder and CBD) and NS IIb (obliteration of gallbladder, CBD, and cystic duct (CD)). Type III is complete atresia involving right and left intrahepatic ducts and extrahepatic ducts [9, 10]. There are many theories of the exact pathophysiology and they include failure of tissue remodeling or abnormal vascular development at the porta hepatis, viral infections, exposure to toxins, and an abnormal immune response leading to ductal obliteration [11]. However, there is still no real evidence as yet to support most of the theories.

There are many technical variants of the Kasai procedure including Type 1BA (cholecystoenterostomy or hepaticoenterostomy), Type 2BA (cystoenterostomy in cases where the hilar cyst communicates with the intrahepatic bile ducts), and Type 3BA (hepatoporto-cholecystostomy) [9, 12]. The procedure vastly enhanced survival and quality of life with many patients surviving into adulthood with their native liver. In addition, the implementation of liver transplant in these patients in the 1980s improved the survival rate immensely.

Overall, the prognosis of patients diagnosed with and treated for biliary atresia has improved significantly for the last 50 years and even approaches 90%. The timing of the Kasai procedure has been investigated and it has been shown that the sooner the procedure is done, the better the outcome of the patient is [1]. Nonetheless, some patients who had a timely procedure to increase exposure of the maximum number of bile ductules, in the porta hepatis, still required transplant [13]. Chardot reviewed all cases of BA in France within 1986–2009. They identified 1107 children of which 1044 underwent Kasai procedure at a median age of 59 days. Five hundred eighty-eight children ultimately underwent 692 liver transplant surgeries. Survival after transplantation was shown to be much higher than survival with native liver (77% versus 30% at 20 years). They concluded that patients who undergo Kasai procedure at an early age require liver transplant at a later age [14].

Factors affecting prognosis include type of BA, state of the liver at the time of surgery, portal pressure, and the association with other malformations. Patients with Types I and II do better as well as those who undergo postoperative resolution of jaundice. It has also been suggested that transplantation should be offered earlier to those patients who do not effectively clear their jaundice [13]. Chardot also reiterated that patients who clear their jaundice after surgery require liver transplantation when they are older as opposed to during infancy or childhood [14]. Patients, in whose explanted livers there are bridging fibrosis and elevated portal pressures at the time of their procedure, tend to do worse postoperatively [1]. It is important to note that many patients still succumb to their disease while waiting on a transplant list.

Furthermore, these patients must be closely monitored for the development of malignancies, which can severely mar survival rate. Hadžić et al. studied 387 infants at King's College Hospital within 1990–2008. Five infants developed HCC within 2–17 years of age. All underwent liver transplantation and were doing well after almost 6 years of follow-up. They suggest that all children with biliary atresia be considered as having the potential to develop HCC. This study concluded that serial alpha-fetoprotein measurements and ultrasonography are still considered the mainstay of early diagnosis [15]. Hol et al. and Aggarwal et al. have suggested the use of repeated sequential magnetic resonance (MR) to monitor the development of liver nodules in this population. Libbrecht et al., in a study of 49 explanted cirrhotic livers, found that ultrasound was poorly sensitive for detecting small hepatocellular carcinomas and suggested contrast-enhanced MR every 6 months in cirrhotic patients. They also cautioned that, oftentimes, benign lesions may be enhanced on the arterial phase and can be mistaken for malignancy and radiologists and pathologists should be aware of this [7].

The management of patients with biliary atresia depends on many factors. These patients need to be diagnosed as early as possible with subsequent timely surgical intervention and close postoperative follow-up. The families of these patients need to realize the necessity of continuity of care and the effect it has on outcome. In addition to close monitoring through imaging, explanted livers of patients with such history as these should be examined and sampled thoroughly, perhaps serially sectioning the liver at 0.5 cm and taking additional samples. In this way, the chances of finding malignant transformation of dysplastic nodules or occult malignancy not demonstrated through imaging are increased.
